# New Methods for Inferring the Distribution of Fitness Effects for INDELs and SNPs

**DOI:** 10.1093/molbev/msy054

**Published:** 2018-04-04

**Authors:** Henry J Barton, Kai Zeng

**Affiliations:** Department of Animal and Plant Sciences, University of Sheffield, Sheffield, United Kingdom

**Keywords:** distribution of fitness effects, insertions and deletions, single nucleotide polymorphism, polarisation error

## Abstract

Small insertions and deletions (INDELs; ≤50 bp) are the most common type of variability after single nucleotide polymorphism (SNP). However, compared with SNPs, we know little about the distribution of fitness effects (DFE) of new INDEL mutations and how prevalent adaptive INDEL substitutions are. Studying INDELs has been difficult partly because identifying ancestral states at these sites is error-prone and misidentification can lead to severely biased estimates of the strength of selection. To solve these problems, we develop new maximum likelihood methods, which use polymorphism data to simultaneously estimate the DFE, the mutation rate, and the misidentification rate. These methods are applicable to both INDELs and SNPs. Simulations show that they can provide highly accurate results. We applied the methods to an INDEL polymorphism data set in *Drosophila melanogaster*. We found that the DFE for polymorphic INDELs in protein-coding regions is bimodal, with the variants being either nearly neutral or strongly deleterious. Based on the DFE, we estimated that 71.5–83.7% of the INDEL substitutions that took place along the *D. melanogaster* lineage were fixed by positive selection, which is comparable with the prevalence of adaptive substitutions at nonsynonymous sites. The new methods have been implemented in the software package anavar.

## Introduction

New mutations can have a range of effects on an organism’s fitness, ranging from being strongly harmful, through being only slightly deleterious, to being neutral, and finally on to being either mildly or highly beneficial. The relative frequencies of mutations with different selective effects is known as the distribution of fitness effects (DFE). The DFE is an important parameter as it is required for addressing many fundamental questions (Eyre-Walker and Keightley 2007). Examples include understanding determinants of the efficacy of natural selection (Galtier 2016; Corcoran et al. 2017), the genetic basis of polygenic traits (Zuk et al. 2014), and the evolutionary advantage of sex and recombination (Hartfield and Keightley 2012).

Taking advantage of the massive increase in data availability, many methods have been proposed for estimating the DFE using polymorphism data (Eyre-Walker et al. 2006; Keightley and Eyre-Walker 2007; Eyre-Walker and Keightley 2009; Kousathanas and Keightley 2013; Kim et al. 2017; Tataru et al. 2017). Their development in turn allows more reliable inferences about other important quantities such as *α*, the proportion of adaptive substitutions (Eyre-Walker and Keightley 2009). However, all these methods are concerned with estimating the DFE for single nucleotide polymorphisms (SNPs). Consequently, much less is known about the DFE and *α* for other types of genetic variation such as small insertions and deletions (INDELs; ≤50 bp), despite the fact that INDELs are the second most common type of variants (e.g., Montgomery et al. 2013), and hence represent an important source of raw materials for selection to act on.

A major difficulty in studying INDELs lies with ancestral state identification. This requires multispecies genome alignments. However, INDELs occur disproportionately in repetitive genomic regions (Ananda et al. 2013; Montgomery et al. 2013), where alignment algorithms perform poorly (Earl et al. 2014). Furthermore, there is evidence that homoplasy is a significant issue outside repetitive regions, probably due to the existence of cryptic INDEL mutation hotspots (Kvikstad and Duret 2014). Thus ancestral state identification can be expected to be particularly error prone for INDELs. It is well established that misidenfication of ancestral states can lead to severely biased estimates of the strength of selection using the site-frequency spectrum (SFS) (Hernandez et al. 2007). For SNPs, this difficulty can be avoided by using the folded SFS (e.g., Eyre-Walker et al. 2006; Keightley and Eyre-Walker 2007). However, to determine whether a length variant is an insertion or a deletion, we have to know what the ancestral state is, meaning that the issue of polarisation error is inherent for INDELs. As a result, applying existing methods for estimating the DFE to INDEL data may be liable to biases.

**Fig. 1.**
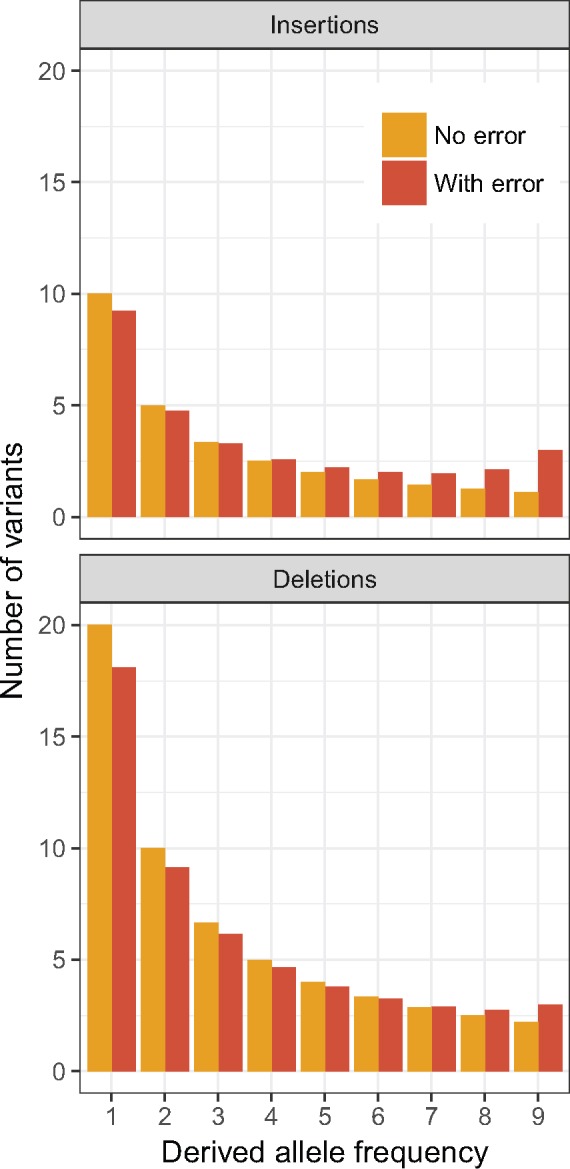
The SFSs for insertions and deletions may be affected to different extents by polarisation errors. We assume that the population size is constant, that INDELs are neutral, and that the sample size is 10. In the genomic region under consideration, the total scaled mutation rate toward insertions, $4N_{e}um$, is 10, where *N_e_* is the effective population size *u* is the insertion mutation rate per site per generation, and *m* is that size of the focal region. The total scaled mutation rate toward deletions is 20. The expected SFSs were generated using standard neutral theory. The SFSs with polarisation errors were generated by assuming that the ancestral state of an INDEL was wrongly identified with probability 0.1.

Another challenge is that the SFSs for insertions and deletions may be affected by polarisation errors to different extents. This is because when the ancestral state of an insertion segregating at low frequency is misidentified, it will be incorrectly inferred as a deletion segregating at high frequency (and vice versa). There is direct experimental evidence that the deletion mutation rate is higher than the insertion mutation rate (Keightley et al. 2009; Schrider et al. 2013; Besenbacher et al. 2015; Yang et al. 2015). This mutational bias means that there are more deletions segregating in the population than insertions. The larger number of deletions may lead to the SFS for insertions being disproportionally affected by polarisation errors (fig. 1). This asymmetry can cause the insertion SFS to have a more pronounced, but artificial, uptick at the high-frequency end, which can be misinterpreted as stronger positive selection on insertions over deletions. As pointed out by Kvikstad and Duret (2014), this methodological issue can, at least in principle, compromises the results of previous studies, which suggest that insertions are more likely to be under positive selection than deletions to prevent the genome size from unconstrained contraction caused by the mutational bias toward deletions (Parsch 2003). Similarly, it will make it difficult to test the possibility that insertions have a higher fixation probability because they are favored by insertion-biased gene conversion (Leushkin and Bazykin 2013).

Toward resolving the confounding efforts ancestral state misidentification have on the study of INDELs, we propose new maximum likelihood methods for inferring the DFE using polymorphism data. These methods are based on recent studies on SNPs which show that polymorphism data contain enough information for simultaneous estimation of the mutation rate, the DFE, and the polarisation error rate (Glémin et al. 2015; Tataru et al. 2017). Our methods are more general than the existing methods in the following aspects. First, they can handle both INDELs and SNPs. Second, insertions and deletions can have different polarisation error rates, mutation rates, and DFEs. Third, for both INDELs and SNPs, the new methods allow the mutation and polarisation error rates to vary across the genome. Incorporating these heterogeneities may be particularly important for INDELs (Kvikstad and Duret 2014). We carried out extensive simulations to examine the performance of the new methods. As an example, we applied the methods to an INDEL polymorphism data set in *Drosophila melanogaster* we obtained by reanalysing the raw short-read data published by the *Drosophila* Population Genomics Project (Pool et al. 2012). Through model comparisons, we tried to find the DFE that best described the observed pattern of INDEL polymorphism within protein-coding regions of the genome. Finally, using the best-fitting DFE, we estimated the proportion of INDEL substitutions fixed by positive selection (*α*).

## New Approach

For ease of presentation, we will start with a description of the SNP models. The INDEL models will be presented later as an extension.

### The SNP Models

Consider a diploid population with effective size *N_e_*. The size of the genomic region of interest is *m* base pairs, and the sample size is *n*.

#### The Discrete Model

Assume that there are *C* different classes of sites in the focal region. These sites can be different with respect to their mutation rates, the fitness effects of new mutations, and polarisation error rates. This discrete model has several advantages. First, it does not assume that the DFE follows a specific probability distribution, and is therefore able to accommodate complex scenarios such as a multimodal DFE (Kousathanas and Keightley 2013). Second, by allowing the mutation and polarisation error rates to vary freely between site classes, the method can include situations whereby these two variables covary (e.g., hypermutable regions may have a higher polarisation error rate).

We assume that the mutation process can be approximated by the infinite-sites model. Let the total scaled mutation rate for sites of class *c* be $m\theta_{c}$, where c∈{1,2,…,C} and $\theta_{c}=4N_{e}u_{c}$. To understand *u_c_*, consider an alternative formulation whereby the mutation rate for the $c^{\text{th}}$ class of sites is *v_c_* per site per generation, and sites of class *c* account for a fraction *p_c_* of all sites in the focal region (i.e., $\sum_{c}p_{c}=1$). We have $m\theta_{c}=mp_{c}4N_{e}v_{c}$, which leads to $u_{c}=p_{c}v_{c}$. By using *θ_c_*, we can perform searches for maximum likelihood estimates (MLEs) of the parameters without having to deal with the constraint $\sum_{c}p_{c}=1$. Define:

(1) $$\theta =\sum_{c=1}^{C}\theta_{c}=4N_{e}\sum_{c=1}^{C}p_{c}v_{c}.$$

Thus, *θ* is the average scaled mutation rate per site, and the total scaled mutation rate is mθ. If the per-site mutation rate is uniform across the focal region (i.e., $v_{i}=v_{j}$ for i≠j and 1≤i,j≤C), then $\theta_{c}/\theta =p_{c}$.

To model selection, we assume that, for mutations arising at sites of class *c*, the fitnesses of the wild-type, heterozygote, and mutant homozygote genotypes are 1, 1 + *s_c_*, and 1 + $2s_{c}$, respectively. The corresponding scaled selection coefficient *γ_c_* is defined as $4N_{e}s_{c}$. Positive and negative *γ_c_* values signify beneficial and deleterious mutations, respectively.

The site-frequency spectrum (SFS) for the *c*^th^ site class, which is defined as the expected number of polymorphic sites of size *i* (i.e., sites where the derived allele is represented *i* times; 1≤i<n), is given by:

(2) $$\Psi_{c,i}=m\theta_{c}\tau_{i}(\gamma_{c})$$

where

(3) $$\tau_{i}(\gamma )=\int_{0}^{1}\left(\begin{array}{c} n\\ i \end{array}\right)x^{i}{\left(1-x\right)}^{n-i}\frac{1-e^{-\gamma (1-x)}}{x(1-x)(1-e^{-\gamma })}dx.$$

Polarisation errors distort the SFS. Specifically, when the ancestral state of a polymorphic site of size *i* is mis-identified, it will be regarded as a polymorphic site of size *n*−*i*. To model polarisation errors, we let *ϵ_c_* be the probability that the ancestral state of a polymorphic site of class *c* is incorrectly identified (Glémin et al. 2015). The final SFS for sites of class *c* is then:

(4) $$\psi_{c,i}=(1-\epsilon_{c})\Psi_{c,i}+\epsilon_{c}\Psi_{c,n-i}.$$

In what follows, we refer to the SFS with and without the correction of polarisation errors as the corrected and uncorrected SFS, respectively. The corrected SFS for the focal region is simply the sum of all the contributions from the sites in different classes:

(5) $$\psi_{i}=\sum_{c=1}^{C}\psi_{c,i}.$$

Existing models either do not model polarisation error (Keightley and Eyre-Walker 2007; Eyre-Walker and Keightley 2009; Kim et al. 2017) or assume that the error rate is constant across the focal region (Glémin et al. 2015; Tataru et al. 2017). The model described here is therefore more general. Allowing variation in the polarisation error rate can be important. For instance, sites under stronger selective constraints tend to evolve slower, and are less likely to be polarised incorrectly due to homoplasy. It should, however, be noted that, when $\gamma_{c}\equiv \gamma$ for ∀c∈{1,2,…,C}, not all the parameters are identifiable. To see this, we rewrite equation (5) as:

(6) $$\psi_{i}=m\sum_{c=1}^{C}\left(1-\epsilon_{c}\right)\theta_{c}\tau_{i}(\gamma )+m\sum_{c=1}^{C}\epsilon_{c}\theta_{c}\tau_{n-i}(\gamma ).$$

Appealing to equation (1) and defining $\epsilon^{*}$ such that

(7) $$\epsilon^{*}\theta =\sum_{c=1}^{C}\epsilon_{c}\theta_{c}$$

we can rewrite equation (6) as

(8) $$\psi_{i}=(1-\epsilon^{*})m\theta \tau_{i}(\gamma )+\epsilon^{*}m\theta \tau_{n-i}(\gamma ).$$

Thus, when there is no difference in fitness effects between mutations arising at sites of different classes, we cannot detect variation in the scaled mutation rate and polarisation error rate because the model reduces to one that depends on *θ*, *γ* and $\epsilon^{*}$. This result has important implications for data analysis by pointing out that a model with a small number of site classes may provide an adequate description of the data even when the underlying biological process features complex variation in the mutation rate across the genome.

#### The Continuous Model

Instead of assuming that the focal region is composed of several classes of sites, we can assume that the fitness effects of new mutations follows a continuous distribution characterised by parameters Ω. Let *θ* be the scaled mutation rate per site, and *ϵ* be the polarisation error rate. The uncorrected SFS becomes:

(9) $$\Psi_{i}=m\theta \int \tau_{i}(\gamma )f(\gamma |\Omega )d\gamma$$

where f(γ|Ω) is the probability density function. The corrected SFS is analogous to equation (4) with *c* in the subscripts omitted.

Although the modeling framework allows the DFE to follow arbitrary probability distribution (including those mixture distributions considered by Galtier 2016), here we only consider the reflected Γ distribution, that is, −γ∼Γ(a,b), where γ≤0 and *a* and *b* are the shape and scale parameters, respectively.

#### Parameter Estimation

Let *X* = (*x*_1_, *x*_2_,…, $x_{n-1}$) represent the observed SFS, where *x_i_* is the number of polymorphic sites of size *i* in the sample. Let Θ denote all the parameters in the model (i.e., *θ_c_*, *γ_c_*, and *ϵ_c_* for c∈{1,2,…,C} for the discrete model and *θ*, Ω, and *ϵ* for the continuous model). To obtain MLEs of Θ, we use the Poisson random field model (Sawyer and Hartl 1992; Bustamante et al. 2001). Omitting constants that have no effects on the shape of the likelihood surface, the log likelihood function is defined as:

(10) $$L(\Theta |X)=\sum_{i=1}^{n-1}(-\psi_{i}+x_{i}\;\text{ln}(\psi_{i})).$$

#### Controlling for Demography

We have so far assumed that the population is panmictic and of constant size *N_e_*. To control for demography, we employ the method of Eyre-Walker et al. (2006). Take the continuous model as an example. First, we define augmented SFSs as:

$$\{\begin{array}{c} \Psi_{i}^{*}=r_{i}\Psi_{i}\;\;\;\;\;\;\;\;\;\;\;\;\;\;\;\;\;\;(11a)\\ \psi_{i}^{*}=(1-\epsilon )\Psi_{i}^{*}+\epsilon \Psi_{n-i}^{*}\;\;\;\;\;\;(11b) \end{array}$$

Next, a set of neutral variants is added to the model, which introduces two additional parameters $\theta^{\left(0\right)}$ and $\epsilon^{\left(0\right)}$, which are the scaled mutation rate per site and the polarisation error rate, respectively, for the neutral sites. Let $\Theta^{\left(0\right)}$ denote these new parameters and $X^{\left(0\right)}$ denote the neutral SFS. The log likelihood of the observed data can be calculated as:

(12) $$L(\Theta ,\Theta^{\left(0\right)},R|X,X^{\left(0\right)})=L(\Theta ,R|X)+L(\Theta^{\left(0\right)},R|X^{\left(0\right)})$$

where *R* = (*r*_2_, *r*_3_,…, $r_{n-1}$) and the two log likelihood functions on the right-hand side are calculated in the same way as equation (10) with *ψ_i_* replaced by $\psi_{i}^{*}$.

The above method for controlling for demography has been used extensively (Eyre-Walker et al. 2006; Muyle et al. 2011; Glémin et al. 2015; Galtier 2016; Jackson et al. 2017; Tataru et al. 2017). These previous efforts have gathered clear theoretical and empirical evidence that the method is robust against a wide range of demographic processes, as well as the effects caused by selection at linked sites (e.g., background selection and/or selective sweeps). For instance, in a recent analysis of selection on codon usage bias in *Drosophila*, Jackson et al. (2017) showed that the estimates of *γ* produced by an estimation method that corrects for demography using the *r* parameters as set out above closely matched those produced by another estimation method that considers an explicit one-step change in population size (see figure 4 A in Jackson et al. 2017).

It should be noted that equation (12) accommodates the possibility that the focal region and the neutral region have different mutation rates. This is more general than several previous models (Keightley and Eyre-Walker 2007; Eyre-Walker and Keightley 2009; Kim et al. 2017; Tataru et al. 2017). However, it may be challenging to distinguish this model from one in which the two regions have the same mutation rate, but a proportion of new mutations in the focal region are so strongly deleterious that they make negligible contributions to the observed SFS.

### The INDEL Models

#### The Discrete Model

First consider insertions. Assume that there are *C^ins^* different classes of sites. The total scaled mutation rate toward insertions for sites of class *c* is $m\theta_{c}^{ins}$, and the fitness effect and polarisation error rate are $\gamma_{c}^{ins}$ and $\epsilon_{c}^{ins}$, respectively ($1\leq c\leq C^{ins}$). The uncorrected SFS for insertions of class *c* can be calculated using equation (2), and is denoted by $\Psi_{c,i}^{ins}$. For deletions, we can similarly assume that there are *C^del^* different classes of sites. The associated parameters are $\theta_{d}^{del},\;\gamma_{d}^{del}$, and $\epsilon_{d}^{del}$, and the uncorrected SFS is denoted by $\Psi_{d,i}^{del}$ ($1\leq d\leq C^{del}$).

When the ancestral state of a derived insertion of size *i* is misidentified, it will be wrongly identified as a deletion of size *n*−*i*, and vice versa for deletions (note that size in this context refers to the frequency of the derived allele, not the number of base pairs inserted or deleted). Thus, the corrected SFSs for insertions and deletions are:

$$\left\{ \begin{array}{c} \psi_{i}^{ins}=\sum_{c=1}^{C^{ins}}\left(1-\epsilon_{c}^{ins}\right)\Psi_{c,i}^{ins}+\sum_{d=1}^{C^{del}}\epsilon_{d}^{del}\Psi_{d,n-i}^{del}\;\;\;\;\;\;\;\;(13a)\\ \psi_{i}^{del}=\sum_{d=1}^{C^{del}}\left(1-\epsilon_{d}^{del}\right)\Psi_{d,i}^{del}+\sum_{c=1}^{C^{ins}}\epsilon_{c}^{ins}\Psi_{c,n-i}^{ins}\;\;\;\;\;\;\;(13b) \end{array}\right.$$

#### The Continuous Model

For insertions, define the per-site scaled mutation rate and the polarisation error rate as *θ^ins^* and *ϵ^ins^*, respectively. The DFE for insertions is determined by parameters $\Omega^{ins}$. For deletions, we similarly define the following parameters: *θ^del^*, $\Omega^{del}$ and *ϵ^del^*. Finally, the corrected SFSs are:

$$\left\{ \begin{array}{c} \psi_{i}^{ins}=(1-\epsilon^{ins})\Psi_{i}^{ins}+\epsilon^{del}\Psi_{n-i}^{del}\;\;\;\;\;\;(14a)\\ \psi_{i}^{del}=(1-\epsilon^{del})\Psi_{i}^{del}+\epsilon^{ins}\Psi_{n-i}^{ins}\;\;\;\;\;(14b) \end{array}\right.$$

where $\Psi_{i}^{ins}$ and $\Psi_{i}^{del}$ are the uncorrected SFSs for insertions and deletions, respectively, and are calculated in the same way as equation (9). As in the SNP case, we only consider cases where the DFE follows a reflected Γ distribution. The shape and scale parameters for insertions and deletions are denoted by *a^ins^*, *b^ins^*, *a^del^*, and *b^del^*, respectively.

#### Parameter Estimation

Let *X^ins^* = ($x_{1}^{ins},\;x_{2}^{ins}$,…, $x_{n-1}^{ins}$) and *X^del^* = ($x_{1}^{del},\;x_{2}^{del}$,…, $x_{n-1}^{del}$) be the observed SFSs for insertions and deletions, respectively. The log likelihood of the data is calculated as:

(15) $$L(\Theta |X^{ins},X^{del})=\sum_{z\in \{ins,del\}}\sum_{i=1}^{n-1}(-\psi_{i}^{z}+x_{i}^{z}\;\text{ln}(\psi_{i}^{z})).$$

#### Controlling for Demography

Take the continuous model as an example. The augmented SFSs are:

$$\left\{ \begin{array}{c} \Psi_{i}^{ins,*}=r_{i}\Psi_{i}^{ins}\;\;\;\;\;\;(16a)\\ \Psi_{i}^{del,*}=r_{i}\Psi_{i}^{del}\;\;\;\;\;(16b)\\ \psi_{i}^{ins,*}=(1-\epsilon^{ins})\Psi_{i}^{ins,*}+\epsilon^{del}\Psi_{n-i}^{del,*\;\;\;\;\;\;\;\;\;\;\;}(16c)\\ \psi_{i}^{del,*}=(1-\epsilon^{del})\Psi_{i}^{del,*}+\epsilon^{ins}\Psi_{n-i}^{ins,*}\;\;\;\;\;\;(16d) \end{array}\right.$$

As for the neutral reference, we can in principle use any combinations of SNPs, insertions, and deletions collected from putatively neutrally evolving regions. Assume that we have access to both neutral insertions and neutral deletions, and the observed SFSs are denoted by $X^{ins,(0)}$ and $X^{del,(0)}$, respectively. The additional parameters needed to model the neutral variants include $\theta^{ins,(0)},\;\epsilon^{ins,(0)},\;\theta^{del,(0)}$, and $\epsilon^{del,(0)}$, which are denoted collectively by $\Theta^{\left(0\right)}$. The log likelihood is:

(17) $$\begin{array}{c} L(\Theta ,\Theta^{\left(0\right)},R|X^{ins},X^{del},X^{ins,(0)},X^{del,(0)})\\ =L(\Theta ,R|X^{ins},X^{del})+L(\Theta^{\left(0\right)},R|X^{ins,(0)},X^{del,(0)}) \end{array}$$

where the two terms on the right are calculated using equation (15) with $\psi_{i}^{z}$ replaced by $\psi_{i}^{z,*}$ (z∈{ins,del}).

## Results and Discussion

### Simulation Results

We evaluate the statistical properties of the new models using computer simulations. Unless stated otherwise, the sample size (*n*) is 50 and the results are based on 100 replicates. In all cases, we assume the population size is constant and only analyse data from the selected region (see Materials and Methods for justification). For the SNP models, we only present results for the discrete SNP model with C>1 site classes, because both the *C* = 1 case and the continuous model have been analysed before (Glémin et al. 2015; Tataru et al. 2017).

#### Properties of the Discrete SNP Model

First consider a model with *C* = 2 site classes. As can be seen from table 1, there is information in the SFS for simultaneously estimating all the parameters to a high degree of accuracy. Before discussing more simulation results, it should be pointed out that, when C>1, the order of the site classes is arbitrary. That is, the model considered in table 1 is equivalent to one with parameters $\theta_{1}=0.01,\;\gamma_{1}=-20,\;\epsilon_{1}=0.01,\;\theta_{2}=0.005,\;\gamma_{2}=-5$, and $\epsilon_{2}=0.05$. For both cases shown in table 1, all the MLEs can be sorted such that ${\hat{\theta }}_{1}<{\hat{\theta }}_{2}$ and ${\hat{\gamma }}_{1}>{\hat{\gamma }}_{2}$. In other words, the MLEs can be assigned unambiguously to site classes according to the order given in the “True value” row. However, if we were to reduce the amount of data, parameter estimates will become more uncertain, and cases such as those with ${\hat{\theta }}_{1}<{\hat{\theta }}_{2}$ and ${\hat{\gamma }}_{1}<{\hat{\gamma }}_{2}$ will occur, which makes assigning the MLEs to site classes impossible. Thus, presenting mean and SD of the MLEs may give misleading information about the performance of the model.

**Table 1.** Maximum Likelihood Estimates (MLEs) of the Parameters of Discrete SNP Models with *C* = 2 Classes of Sites.

	*m*	$\theta_{1}$	$\gamma_{1}$	$\epsilon_{1}$	$\theta_{2}$	$\gamma_{2}$	$\epsilon_{2}$
True value	–	0.005	−5	0.05	0.01	−20	0.01
Mean (SD) of MLEs	10^6^	0.0050 (0.0007)	−5.0 (0.4)	0.051 (0.006)	0.010 (0.001)	−20.2 (1.9)	0.009 (0.006)
Mean (SD) of MLEs	10^5^	0.0044 (0.0017)	−4.4 (1.5)	0.042 (0.022)	0.011 (0.001)	−20.0 (5.7)	0.016 (0.014)

Note.—Simulated data were generated using the parameter values shown in the “True value” row, with two different region sizes, *m*. For each parameter combination, 100 samples of size 50 were simulated and analysed to obtain MLEs.

In light of the earlier discussion, we investigate the statistical properties of the model using two alternative methods. First, we compare the full model to the following reduced models using the χ^2^ test: “Equal *ϵ*” (all site share the same polarisation error rate), “*ϵ* = 0” (no polarisation error), and “C−1”(a model with C−1 site classes, where *C* is the true number of site classes). Second, we assess how well these various models predict the average fixation probability $\bar{\mu }$ (see eq. 18 in Materials and Methods), which is essential for estimating the prevalence of adaptive substitutions (i.e., *α* and *ω_a_*).

Considering the two pairs of cases in table 2, and focusing on the data presented under “Percent significant,” we make the following observations. First, as the amount of data reduces, the ability of the model to infer separate *ϵ* for different site classes drops more rapidly than its ability to detect the existence of either polarisation error or more than one site class. This suggests that estimating heterogeneity in *ϵ* may be challenging. Considering all four cases, it appears that the tests for detecting the presence of polarisation error (i.e., the full model vs. “*ϵ* = 0”) and for detecting the existence of more site classes (i.e., the full model vs. “C−1”) are more powerful, especially the latter. It should be noted that the likelihood surface appears to be rather flat when *C* = 3 such that different parameter combinations may produce very similar log likelihoods. This is particularly evident when the amount of data is limited (Case 3 vs. Case 4), leading to a reduction in power of the tests. A similar observation was made by Keightley and Eyre-Walker (2010), who also showed that it can be partly alleviated by increasing the sample size. Nonetheless there may well be a limit as to how many site classes can be included. This identifiability problem is analogous to that discussed extensively in the context of using SNP-based methods for estimating past demographic changes (e.g., Myers et al. 2008).

**Table 2.** Statistical Properties of the Discrete SNP Model.

Case	Parameters	*m*	Percent Significant			$\bar{\mu }$				
			Equal *ϵ*	*ϵ* = 0	C−1	True	Full	Equal *ϵ*	*ϵ* = 0	C−1
1	Same as table 1	10^6^	93	100	100	0.0113	0.0114	0.0171	>1	0.0022
2	Same as table 1	10^5^	15	92	100	0.0113	0.0158	0.0204	>1	0.0022
3	See notes below	10^7^	3	100	100	0.2204	0.2267	0.2613	>1	0.1755
4	Same as Case 3	$2\times 10^{6}$	0	33	55	0.2204	0.2271	0.2580	>1	0.1768

Note.—The parameters used in Case 3 were $\theta_{1}=0.002,\;\gamma_{1}=0,\;\epsilon_{1}=0.05,\;\theta_{2}=0.006,\;\gamma_{2}=-5,\;\epsilon_{2}=0.02,\;\theta_{3}=0.002,\;\gamma_{3}=-30,\;\epsilon_{3}=0.01$, and *n* = 100. A large sample size was used for Cases 3 and 4 due to the inclusion of strongly deleterious mutations (i.e., $\gamma_{3}=-30$). Values under “Percent significant” show how often the full model fitted the data better than the three reduced models (see the main text for more details). The $\bar{\mu }$ (see eq. 18 in Materials and Methods) obtained under the *ϵ* = 0 model are large because ignoring polarisation error results in the inference of a site class with a strongly positive *γ*.

Interestingly, the reduced model “Equal *ϵ*” makes worse predictions of $\bar{\mu }$ than the full model in all cases presented in table 2, even when the full model does not normally provide a better fit to the data (Cases 2 and 4). The same applies to the other two reduced models. Thus, despite the statistical difficulties discussed earlier, fitting the full model to the data may be important for obtaining accurate estimates of *α* and *ω_a_*.

#### Properties of the INDEL Models

Table 3 contains simulation results based on a discrete model (with $C^{ins}=C^{del}=1$) and two continuous models (differing from each other in terms of the size of the focal region *m*). The mutation rates are ∼10 times lower than those used in the SNP cases (tables 1 and 2), and polarisation error rates are ∼2 times higher. These choices are to reflect the fact that INDELs are generally less prevalent than SNPs, and are potentially more difficult to polarise. As can be seen, with a reasonable amount of data, all the parameters can be reliably estimated. Comparing the two continuous models, we notice that, with limited data, the scale parameter *b* of the Γ distribution may be overestimated, but estimates of the shape parameter *a* and the polarisation error rate remain unbiased.

**Table 3.** MLEs of the Parameters of Several INDEL Models.

Model	*m*	Parameters								
Discrete	$2\times 10^{6}$	Name	$\theta_{1}^{ins}$	$\gamma_{1}^{ins}$	$\epsilon_{1}^{ins}$	$\theta_{1}^{del}$	$\gamma_{1}^{del}$	$\epsilon_{1}^{del}$		
		True	0.0005	−5	0.02	0.001	−15	0.02		
		Mean MLE	0.00050	−5.0	0.021	0.0010	−15.0	0.020		
Continuous	$2\times 10^{7}$	Name	*θ^ins^*	*a^ins^*	*b^ins^*	*ϵ^ins^*	*θ^del^*	*a^del^*	*b^del^*	*ϵ^del^*
		True	0.0005	0.5	10	0.08	0.001	0.25	50	0.04
		Mean MLE	0.00050	0.51	10.4	0.080	0.0010	0.251	51.2	0.040
Continuous	$2\times 10^{6}$	Name	*θ^ins^*	*a^ins^*	*b^ins^*	*ϵ^ins^*	*θ^del^*	*a^del^*	*b^del^*	*ϵ^del^*
		True	0.0005	0.5	10	0.08	0.001	0.25	50	0.04
		Mean MLE	0.00054	0.51	144.7	0.082	0.0010	0.253	93.2	0.041

The true values of ${\bar{\mu }}^{ins}$ and ${\bar{\mu }}^{del}$ for the discrete model are 0.0339 and $4.59\times 10^{-6}$, respectively. The mean (SD) of the estimates is 0.0345 (0.0055) for ${\bar{\mu }}^{ins}$, and $5.27\times 10^{-6}$ ($2.91\times 10^{-6}$) for ${\bar{\mu }}^{del}$. Thus, the true values are well within the observed ranges of variability. The true values of ${\bar{\mu }}^{ins}$ and ${\bar{\mu }}^{del}$ for the two continuous cases are 0.384 and 0.429, respectively. The mean (SD) of the estimates for the case with more data is 0.382 (0.012) for ${\bar{\mu }}^{ins}$ and 0.429 (0.008) for ${\bar{\mu }}^{del}$. Encouragingly, for the continuous case with less data, despite the tendency to overestimate the scale parameter, estimates of the average fixation probabilities are still highly accurate: 0.388 (0.050) for ${\bar{\mu }}^{ins}$ and 0.418 (0.028) for ${\bar{\mu }}^{del}$, suggesting that the reliability of estimates of *α* and *ω_a_* is unlikely to be compromised.

### Application to *D. melanogaster* Data

#### A Summary of the Data

Using the variant calling pipeline detailed in Materials and Methods, a total of 370, 217 INDELs (≤50 bp) and 1,789,367 SNPs were identified from the 17 Rwandan individuals. Our analysis primarily focuses on INDELs because SNPs have been analysed extensively before (Keightley and Eyre-Walker 2007; Eyre-Walker and Keightley 2009; Schneider et al. 2011). Similar to previous reports (e.g., Ptak and Petrov 2002), smaller INDELs are more prevalent than larger ones (supplementary fig. S1, Supplementary Material online). INDEL diversity is ∼30 times lower in protein-coding (CDS) regions than in either intronic or intergenic regions (table 4). Additionally, frameshift INDELs are rarer than nonframeshift ones (table 4 and supplementary fig. S1, Supplementary Material online). Interestingly, nonsense mutations are somewhat rarer than frameshift INDELs, an observation also made by Leushkin et al. (2013). These results indicate strong purifying selection against INDELs in protein-coding regions. INDEL diversity patterns appear to be similar between intronic and intergenic regions. They are combined and referred to as noncoding INDELs in what follows to increase statistical power.

**Table 4.** Summary Statistics for the INDEL and SNP Data.

Data	Type	Diversity (*π*)	Tajima’s *D*
INDELs	CDS	$5.20\times 10^{-5}$	−1.208
	Frameshift	$2.06\times 10^{-5}$	−1.253
	Nonframeshift	$3.14\times 10^{-5}$	−1.177
	Intron	0.0016	−0.729
	Intergenic	0.0017	−0.704
	Noncoding	0.0017	−0.718
SNPs	Nonsense	$5.83\times 10^{-6}$	−1.510
	0-fold degenerate sites	0.0016	−0.868
	4-fold degenerate sites	0.0165	−0.210

Comparing between INDELs and SNPs, we notice that INDEL diversity in noncoding regions is ∼10 times lower than $\pi_{4}$ (4-fold site diversity; table 4), consistent with the fact that the INDEL mutation rate is lower than the point mutation rate (Haag-Liautard et al. 2007; Schrider et al. 2013). However, Tajima’s *D* calculated on noncoding INDELs is more negative than that calculated on 4-fold sites (table 4), probably reflecting the fact that many noncoding DNA in the *D. melanogaster* genome are under selection (Andolfatto 2005). Furthermore, $\pi_{0}$ (0-fold site diversity; table 4) is only ∼10 times smaller than $\pi_{4}$. This level of reduction is much smaller than the 30-fold difference observed between CDS and noncoding INDELs. This suggests that, in protein-coding regions, INDEL mutations are under much stronger purifying selection than 0-fold mutations, which is consistent with the more negative Tajima’s *D* value calculated on CDS INDELs (table 4).

To further investigate the data, we calculated *d*_N_, substitution rate at nonsynonymous sites, using PAML and the reference genomes of *D. simulans* and *D. yakuba* (see Materials and Methods). The genes were then divided into 20 equal-sized bins. For each bin, we calculated average $\pi_{0}$ and $\pi_{\text{INDEL}}$. Both statistics decrease as *d*_N_ decreases (supplementary fig. S2, Supplementary Material online), consistent with the expectation that mutations are on average more deleterious in more conserved genes (Jackson et al. 2015). The results in this and the preceding paragraphs suggest that our INDEL data set is of high quality.

#### Inferring the DFE and α Using Noncoding INDELs as the Neutral Reference

To infer the DFE for INDELs in CDS regions, we used noncoding INDELs as the neutral reference. Following previous efforts in estimating the DFE for SNPs (Keightley and Eyre-Walker 2007; Eyre-Walker and Keightley 2009; Schneider et al. 2011; Galtier 2016; Tataru et al. 2017), we also assumed that the mutation rate toward insertions and deletions, respectively, were the same between the neutral and selected regions. The best-fitting DFE is one with *C* = 2 classes of selected sites (table 5 and supplementary table S1, Supplementary Material online). The MLEs of *γ* suggest that polymorphic INDELs are either nearly neutral or are so strongly deleterious that they contribute little to polymorphism. This seems to be consistent with the 30-fold difference in INDEL diversity level between CDS and noncoding regions, which is more substantial than the 10-fold difference between 0-fold and 4-fold sites (table 4). Fitting the data to a discrete model with *C* = 3 classes of sites also reveals a bimodal DFE, suggesting that the conclusion is robust (supplementary table S1, Supplementary Material online). With a larger sample containing hundreds or even thousands of alleles, and by fitting a DFE with more site classes, it should be possible to obtain further details of the relative frequencies and fitness effects of strongly selected variants, which tend not to segregate in our current sample of size 17. However, this additional information about the strongly selected end of the DFE is unlikely to affect our estimation of *α* (see below) because these variants make effectively no contribution to divergence.

**Table 5.** Results Based on the Best-Fitting Models for INDELs in the CDS Regions of the *D. melanogaster* Genome.

Neutral Ref/DFE/Mutation Rate	Parameters for CDS INDELs							*α*
Noncoding INDELs	Name	$\theta_{1}^{ins}$	$\gamma_{1}^{ins}$	$\epsilon_{1}^{ins}$	$\theta_{1}^{del}$	$\gamma_{1}^{del}$	$\epsilon_{1}^{del}$	83.7%
Discrete *C* = 2	MLE	$1.8\times 10^{-5}$	1.98	0.023	$5.3\times 10^{-5}$	−1.69	0.016	
Uniform mutation rate	Name	$\theta_{2}^{ins}$	$\gamma_{2}^{ins}$	$\epsilon_{2}^{ins}$	$\theta_{2}^{del}$	$\gamma_{2}^{del}$	$\epsilon_{2}^{del}$	
	MLE	$7.2\times 10^{-4}$	−1566.4	$3.6\times 10^{-5}$	0.0011	−642.5	$1.6\times 10^{-5}$	
4-fold degenerate sites	Name	$\theta_{1}^{ins}$	$\gamma_{1}^{ins}$	$\epsilon_{1}^{ins}$	$\theta_{1}^{del}$	$\gamma_{1}^{del}$	$\epsilon_{1}^{del}$	71.5%
Discrete *C* = 2	MLE	$1.6\times 10^{-5}$	−1.31	0.0092	$4.9\times 10^{-5}$	−3.77	0.0082	
Fixed mutation ratios	Name	$\theta_{2}^{ins}$	$\gamma_{2}^{ins}$	$\epsilon_{2}^{ins}$	$\theta_{2}^{del}$	$\gamma_{2}^{del}$	$\epsilon_{2}^{del}$	
	MLE	$1.9\times 10^{-4}$	−284.1	$1.2\times 10^{-4}$	0.0010	−454.8	$6.2\times 10^{-5}$	

Note.—The DFE for polymorphic INDELs in the CDS regions were inferred using either noncoding INDELs or 4-fold sites as the neutral reference. A series of different DFEs were fitted to the data, and the best-fitting models presented above were determined by using the Akaike information criterion (AIC) (see supplementary tables S1 and S5, Supplementary Material online). When noncoding INDELs were used as the neutral reference, *α* was estimated using INDEL divergence in noncoding regions. When 4-fold sites were used as the neutral reference, the mutation rate ratio between SNPs and INDELs, and that between deletions and insertions, were fixed at values obtained from a mutation accumulation experiment (Schrider et al. 2013). *α* was estimated using a method based on divergence in the 8–30 bp region of short introns < 66 bp long (see the main text).

To better understand the effects of length, we separated the INDELs in CDS regions into the following length categories: 1 bp, 2 bp, 3 bp, frameshifting (≥4 bp), and nonframeshifting (≥6 bp). We analysed the data in each category separately. As above, noncoding INDELs with the same length were used as the neutral reference and the mutation was assumed to be constant across neutral and selected sites. Considering the dearth of variants, we only fitted a DFE with *C* = 1 class of selected sites. Viewing the *γ* in this model as the “average” selection coefficient, frameshift INDELs are consistently more deleterious than nonframeshift INDELs (supplementary fig. S3, Supplementary Material online). Consistent with a prevous study (Leushkin et al. 2013), there is no obvious evidence that longer INDELs are under stronger selection.

Using the best-fitting DFE (table 5), the proportion of INDEL substitutions in the CDS regions fixed by positive selection in the *D. melanogaster* lineage, *α*, is 83.7% (100% for insertions and 81.8% for deletions). These *α* estimates are comparable with previous estimates for SNP substitutions in CDS regions (Andolfatto et al. 2011; Schneider et al. 2011).

As mentioned earlier, some noncoding INDELs are probably nonneutral, as suggested by the negative Tajima’s *D* value (table 4). Our use of these variants as the neutral reference are for several practical reasons. Although using INDELs in “dead-on-arrival” transposable elements as neutral reference may be preferable (Petrov 2002), calling variants from repetitive regions using short-read data is highly prone to error (Li 2014). Using data from the 8–30 bp region of short introns ≤65 bp, which are also putatively neutral (Parsch et al. 2010), is also problematic because of evidence for selection maintaining intron size (Ptak and Petrov 2002; Parsch 2003; Leushkin et al. 2013). Note that Tajima’s *D* is more negative for INDELs in CDS regions than for those in noncoding regions, suggesting that the latter are probably under weaker purifying selection (table 4). If this is the case, our method tends to underestimate the strength of purifying selection on INDELs in CDS regions, as suggested by the simulation results presented in supplementary table S2, Supplementary Material online. This should lead to an overestimation of $\bar{\mu }$, the average fixation rate (eq. 18), which should in turn put a downward pressure on the estimation of *α* (eq. 19). However, biases in *α* also depend on the way selection on noncoding INDELs alters divergence. For example, if fixations of beneficial noncoding INDELs are so common that *d*_S_ is greater than the divergence level expected under neutral evolution, then this combined with the overestimation of $\bar{\mu }$ can lead to a substantial underestimation of *α*. In contrast, if most noncoding INDELs are selected against and *d*_S_ is much smaller than the neutral expectation, it may offset the effect caused by the overestimation of $\bar{\mu }$ and result in an overestimation of *α*.

#### Inferring the DFE and α Using 4-Fold Degenerate Sites as the Neutral Reference

To check the robustness of our results, we conducted a second set of analyses without using noncoding INDELs. We extended our model such that it can infer the DFE for INDELs in CDS regions using 4-fold sites as the neutral reference. We chose 4-fold sites instead of the 8–30 bp region of short introns ≤65 bp because 4-fold sites are probably not under ongoing selection on codon usage in *D. melanogaster*, and are similar to short introns in multiple aspects of polymorphism patterns (Jackson et al. 2017). Considering the parameter richness of the models, using 4-fold SNPs as the neutral reference should help statistical inference because they are much more numerous than short-intron SNPs.

We used the following approach to obtain neutral divergence for INDELs along the *D. melanogaster* lineage. The nucleotide divergence in the 8–30 bp region of short introns ≤65 bp is 0.0674 (Jackson B, personal communication). In a mutation accumulation experiment (Schrider et al. 2013), it was found that the rate to point mutations is 12.2 times higher than that to short INDELs, and that the rate to deletions is 5 times higher than that to insertions (averaging across the two genetic backgrounds considered therein). Thus, an estimate of neutral INDEL divergence can be obtained as 0.0674/12.2=0.0055, and the corresponding estimates for insertions and deletions are $9.2\times 10^{-4}$ and 0.0046, respectively.

Due to the use of 4-fold sites as the neutral reference, it is no longer appropriate to assume that the mutation rate is the same between the selected and neutral regions. Given the evidence that the DFE for INDELs probably features a class of strongly deleterious mutations that make little contribution to polymorphism, allowing the selected and neutral regions to have their separate mutation rates is likely to cause the model to underestimate both the mutation rate in the selected region and strength of purifying selection, as confirmed by simulation results presented in supplementary table S3, Supplementary Material online. An underestimation of the strength of purifying selection is likely to cause an underestimation of *α*. We observed this in our data set—*α* for all INDELs obtained from the best-fitting DFE for this analysis (supplementary table S4, Supplementary Material online) is only 21.7%, much smaller than the value of 83.7% when noncoding INDELs were used as the neutral reference (table 5).

To resolve the above problem, we again made use of the information reported in the aforementioned mutation accumulation experiment (Schrider et al. 2013). Specifically, we further extended our model, so that the mutation rate ratio between SNPs and INDELs, and that between deletions and insertions, were fixed at 12.2 and 5, respectively. As shown in table 5 (see also supplementary table S5, Supplementary Material online), the best-fitting DFE has *C* = 2 class of sites, with one under weak selection, and the other being strongly deleterious. The *α* estimates for all INDELs, insertions, and deletions are, respectively, 71.5%, 59.7%, and 81.3%.

To make sure that the above results are not dependent on our use of the mutation rate ratios estimated by Schrider et al. (2013), we repeated the analysis using ratios obtained by either Petrov and Hartl (1998) (SNP/INDEL = 6.9 and deletion/insertion = 8.7) or Haag-Liautard et al. (2007) (SNP/INDEL = 4.2 and deletion/insertion = 3.0) (supplementary table S6, Supplementary Material online). In both cases, the best-fitting DFE has *C* = 2 classes of selected sites, under weak and strong selection, respectively (supplementary tables S7 and S8, Supplementary Material online). Furthermore, estimates of the strength of purifying selection acting on sites in the weakly selected class are almost identical regardless of the choice of mutation rate ratios (supplementary table S9, Supplementary Material online). Thus, unsurprisingly, all three analyses also produce very similar *α* estimates (supplementary table S9, Supplementary Material online). Overall, these results are consistent with those based on noncoding INDELs and suggest that a substantial fraction of INDEL substitutions were fixed by positive selection.

## Materials and Methods

### Numerical Details

We used numerical routines provided by the GNU Scientific Library (GSL; https://www.gnu.org/software/gsl/; last accessed April 6, 2018) to perform the integration in equation (3) numerically. For the continuous model (e.g., eq. 9), the integral was evaluated using Gaussian quadrature, which was implemented based on a routine included in the R package statmod (https://cran.r-project.org/web/packages/statmod/index.html; last accessed April 6, 2018). Maximum likelihood estimates of the model parameters were obtained by both gradient-based and derivative-free optimization algorithms implemented in the NLopt package (http://ab-initio.mit.edu/wiki/index.php/NLopt; last accessed April 6, 2018). To ensure the global maximum was found, we initialised the search algorithm using multiple randomly selected starting points.

### Simulations

We performed parameter estimation using our program, anavar, on random samples simulated using Mathematica (http://www.wolfram.com/; last accessed April 6, 2018). Because the generation of simulated data is separate from the numerical routines we used to implement anavar, this set-up can help verify the numerical robustness of anavar. Note that, in all simulations, we only used the models to analyse variants from selected regions because we wanted to find out how much information we could obtain by analysing them alone. Including neutral variants, as routinely done in real data analysis, may help to increase the accuracy of parameter estimation. Therefore, our choice should give us a rather conservative assessment of the methods’ performance.

In addition to testing whether the data contained enough information for all the parameters to be estimated, we also assessed how well a model could predict the average fixation rate, $\bar{\mu }$ (expressed in units of $2N_{e}$ generations). As an example, if nonsynonymous polymorphism data are fitted to the discrete SNP model, $\bar{\mu }$ can be estimated as:

(18) $$\bar{\mu }=\frac{1}{\hat{\theta }}\sum_{c=1}^{C}\frac{{\hat{\theta }}_{c}{\hat{\gamma }}_{c}}{1-e^{-{\hat{\gamma }}_{c}}}$$

where $\hat{Z}$ signifies the MLE of parameter *Z* and *θ* is defined by equation (1). Understanding the ability to accurately estimate $\bar{\mu }$ is important because it is needed for estimating *α*, the proportion of substitutions fixed by positive selection, which can be written as:

(19) $$\alpha =\frac{d_{N}-d_{S}\bar{\mu }}{d_{N}}$$

where *d*_N_ and *d*_S_ are the numbers of selected (e.g., nonsynonymous) and neutral (e.g., synonymous) substitutions per site, respectively (Eyre-Walker and Keightley 2009).

We did not generate simulated data from models with demographic changes and selection at linked sites because the effectiveness of the method of Eyre-Walker et al. (2006) in controlling for these confounding factors have been studied extensively (Eyre-Walker et al. 2006; Muyle et al. 2011; Glémin et al. 2015; Galtier 2016; Jackson et al. 2017; Tataru et al. 2017).

### The *Drosophila melanogaster* Data Set

This data set consisted of 17 Rwandan individuals as described in Jackson et al. (2015, 2017) and made available by the *Drosophila* Population Genomics Project (Pool et al. 2012).

#### Variant Calling

INDEL realigned BAM files were obtained from Jackson et al. (2017). Initial genotype calling was performed with the HaplotypeCaller and GenotypeGVCF (with the -includeNonVariantSites flag to output genotype calls at both variant and nonvariant positions) tools from GATK 3.7 (DePristo et al. 2011; Van der Auwera et al. 2013). Variant quality score recalibration (VQSR) requires one “truth set” for SNPs and one for INDELs. To generate the truth sets, we intersected the raw variants called from GATK with variants called from SAMtools (version 1.2) (Li et al. 2009). The consensus data were further filtered using the GATK best practice hard filters (for SNPs: QD < 2.0, MQ < 40.0, FS > 60.0, SOR > 3.0, MQRankSum < −12.5, ReadPosRankSum < −8.0; for INDELs: QD < 2.0, ReadPosRankSum < −20.0, FS > 200.0, SOR > 10.0; see https://software.broadinstitute.org/gatk/guide/article? id=3,225; last accessed April 6, 2018). Variants with coverage more than twice, or less than half, the mean coverage of 20× were excluded, along with variants falling into regions identified by RepeatMasker (http://www.repeatmasker.org). Multiallelic sites were excluded along with SNPs falling within INDELs and INDELs >50 bp. We ran VQSR separately for SNPs and INDELs, retaining variants that fell within the 95% tranche cut-off as in Jackson et al. (2017). The passing variants were then refiltered as above with the exception of the GATK hard filters which were not reapplied.

#### Multispecies Alignments and Polarisation

Multispecies alignments were generated between *D. melanogaster* (v5.34), *D. simulans* (Hu et al. 2013), and *D. yakuba* (v1.3) using *D. melanogaster* as reference. Firstly pairwise alignments were created using LASTZ (Harris 2007). These were then chained and netted using axtChain and chainNet, respectively (Kent et al. 2003). Single coverage was ensured for the reference genome using single_cov2.v11 from the MULTIZ package (Blanchette et al. 2004) and the pairwise alignments were aligned with MULTIZ.

Variants were polarised using the whole genome multispecies alignment and a parsimony approach, where either the alternate or the reference allele had to be supported by all outgroups in the the alignment to be considered ancestral. The site-frequency spectra for insertions and deletions in different genomic regions are presented in supplementary figure S4, Supplementary Material online.

#### Annotation

Variants were annotated as either intronic, intergenic, or CDS using the *D. melanogaster* GFF annotation file (version 5.34, available from: ftp://ftp.flybase.net/genomes/Drosophila_melanogaster/dmel_r5.34_FB2011_02/gff/). Four-fold degenerate and 0-fold degenerate SNPs in CDS regions were annotated using coordinates obtained from the *D. melanogaster* CDS fasta sequences (version 5.34, available from: ftp://ftp.flybase.net/genomes/Drosophila_melanogaster/dmel_r5.34_FB2011_02/fasta/dmel-all-CDS-r5.34.fasta.gz).

#### Summary Statistics

Nucleotide diversity (*π*) (Tajima 1983), Watterson’s *θ* (Watterson 1975), and Tajima’s *D* (Tajima 1989) were calculated for variants in noncoding (intronic and intergenic) and coding regions, as well as for 0-fold and 4-fold degenerate SNPs. The numbers of callable sites used to obtain per-site estimates was taken to be the number of sites in each region that were called in the “all sites” VCF file and passed the filters described previously. Additionally for polarised variants the number of callable sites was reduced to those that could be polarised by our parsimony approach.

To obtain rates of divergence at nonsynonymous and synonymous sites, denoted by *d*_N_ and *d*_S_, CDS regions were extracted from the multispecies alignment using the coordinates from the *D. melanogaster* CDS fasta alignment file. CDS alignments were removed if they were not in frame, did not start with a start codon, did not end with a stop codon or contained premature stop codons. Additionally any codons with missing data were dropped. For each gene, we retained only the longest transcript. These data were then analysed using codeml in PAML (Yang 2007) with a one ratio model to obtain *d*_N_ and *d*_S_. 

#### Software Availability

The new models have been implemented in a user-friendly package anavar, which is freely available at http://zeng-lab.group.shef.ac.uk. In addition to the models developed herein, anavar also contains implementations of several other widely-used models for estimating the DFE (Eyre-Walker et al. 2006) and for studying GC-biased gene conversion (gBGC) (Glémin et al. 2015). All scripts used for the anavar simulation analyses are available at https://github.com/henryjuho/anavar_simulations. Additionally, all scripts used in the *D. melanogaster* analyses can be found at https://github.com/henryjuho/drosophila_indels.

## Supplementary Material

Supplementary data are available at *Molecular Biology and Evolution* online.

## Supplementary Material

Supplementary Data
