# Supplementary material for: New Methods for Inferring the Distribution of Fitness Effects for INDELs and SNPs
Source: Mol Biol Evol. 2018 Apr 4;35(6):1536–46. doi: 10.1093/molbev/msy054 (PMC5967470; doi:10.1093/molbev/msy054)
Supplement: Supplementary Data [file msy054_supp.zip › supplement_v2.docx]

**Figure S1.** Length distribution of INDEL polymorphism in *D. melanogaster*. Upper panel: the distribution of all INDELs called (≤ 50bp). Lower panel: the distribution of INDELs within CDS regions.

**Figure S2.** The correlation between *d_N_* and *π*_0_ or *π*_INDEL_. The genes were binned based on *d_N_* into 20 equal-sized groups.

**Figure S3.** Estimates of *γ* for polymorphic INDELs of different lengths. The INDELs in the *D. melanogaster* dataset were divided into the following length categories: 1bp, 2bp, 3bp, frameshifting (≥ 4bp), non-frameshifting (≥ 6bp). Non-coding INDELs with the same lengths were used as the neutral reference. The data were analysed using a model with 1 class of sites in the selected region and uniform mutation rate across the selected and neutral regions.

**Figure S4.** The site-frequency spectra for insertions and deletions in different genomic regions in the *D. melanogaster* dataset.

**Table S1**. Results based on fitting the new models to the INDELs within protein-coding regions from *D. melanogaster*. Non-coding INDELs were used as the neutral reference, and the mutation rates to insertions and deletion were assumed to be equal between selected and neutral regions.

| Model | Parameters for INDELs in the CDS regions | | | | | | | | | ΔAIC |
| --- | --- | --- | --- | --- | --- | --- | --- | --- | --- | --- |
| Discrete | Name | ${\theta_{1}}^{ins}$ | ${\gamma_{1}}^{ins}$ | ${\epsilon_{1}}^{ins}$ | ${\theta_{1}}^{del}$ | ${\gamma_{1}}^{del}$ | ${\epsilon_{1}}^{del}$ |  |  | 0 |
| *C*=2 | MLE | 1.8 × 10^−5^ | 1.98 | 0.023 | 5.3 x 10^-5^ | -1.69 | 0.016 |  |  |  |
|  | Name | ${\theta_{2}}^{ins}$ | ${\gamma_{2}}^{ins}$ | ${\epsilon_{2}}^{ins}$ | ${\theta_{2}}^{del}$ | ${\gamma_{2}}^{del}$ | ${\epsilon_{2}}^{del}$ |  |  |  |
|  | MLE | 0.0007 | -1566.4 | 3.6 x 10^-5^ | 0.0011 | -642.5 | 1.6 × 10^-5^ |  |  |  |
| Discrete | Name | ${\theta_{1}}^{ins}$ | ${\gamma_{1}}^{ins}$ | ${\epsilon_{1}}^{ins}$ | ${\theta_{1}}^{del}$ | ${\gamma_{1}}^{del}$ | ${\epsilon_{1}}^{del}$ |  |  | 9 |
| *C*=3 | MLE | 1.86 × 10^−5^ | 1.41 | 0.022 | 5.5 × 10^−5^ | -3.03 | 0.0040 |  |  |  |
|  | Name | ${\theta_{2}}^{ins}$ | ${\gamma_{2}}^{ins}$ | ${\epsilon_{2}}^{ins}$ | ${\theta_{2}}^{del}$ | ${\gamma_{2}}^{del}$ | ${\epsilon_{2}}^{del}$ |  |  |  |
|  | MLE | 0.0002 | -657.5 | 0.0003 | 0.0011 | -771.9 | 2.4 x 10^-5^ |  |  |  |
|  | Name | ${\theta_{3}}^{ins}$ | ${\gamma_{3}}^{ins}$ | ${\epsilon_{3}}^{ins}$ | ${\theta_{3}}^{del}$ | ${\gamma_{3}}^{del}$ | ${\epsilon_{3}}^{del}$ |  |  |  |
|  | MLE | 0.0005 | -4584.3 | 1.5 x 10^-5^ | 5.1 x 10^-6^ | 885.9 | 0.169 |  |  |  |
| Continuous | Name | $\theta^{ins}$ | $\alpha^{ins}$ | $b^{ins}$ | $\epsilon^{ins}$ | $\theta^{del}$ | $\alpha^{del}$ | $b^{del}$ | $\epsilon^{del}$ | 484 |
|  | MLE | 0.0007 | 0.71 | 2596.7 | 0.033 | 0.0012 | 0.63 | 2417.1 | 0.038 |  |
| Discrete | Name | ${\theta_{1}}^{ins}$ | ${\gamma_{1}}^{ins}$ | ${\epsilon_{1}}^{ins}$ | ${\theta_{1}}^{del}$ | ${\gamma_{1}}^{del}$ | ${\epsilon_{1}}^{del}$ |  |  | 6831 |
| *C*=1 | MLE | 0.0007 | -96.3 | 0.096 | 0.0011 | -70.0 | 0.054 |  |  |  |

**Table S2.** The effects of the presence of selected variants in the neutral reference on the estimation of the strength of selection on variants in the selected dataset. The sample size is 17 in all cases, the mutation rate is uniform across the genome, and the polarisation error rate is zero. The “neutral” reference dataset was generated with *γ* = -3.5. The selected dataset was generated using three different *γ* values. The simulated data were analysed by a model that considers a single class of selected sites, involves the use of the *r* parameters (see Eq. (11)), and assumes a uniform mutation rate. The results are based on 50 replicates. $\overline{\mu}$ is the average fixation probability.

| *γ* for selected variants | | $\overline{\mu}$ | |
| --- | --- | --- | --- |
| True | Mean(MLEs) | True | Mean(MLEs) |
| -1.5 | 6.997 | 0.431 | 7.004 |
| -3.5 | -0.018 | 0.109 | 0.992 |
| -10 | -7.268 | 4.5 × 10^-4^ | 0.005 |

**Table S3.** Simulations showing the effects of strong purifying selection parameter estimations when the neutral and selected regions are allowed to have separate mutation rate parameters. The sample size is 17, and the results are based on 50 replicates. The data were simulated by assuming that the DFE follows a reflected gamma distribution with different shape (*a*) and scale (*b*) parameters. Note that the mean for *γ* is $\bar{\gamma}$ = –*ab*. *θ* is the scaled mutation rate per site. $\overline{\mu}$ is the average fixation probability.

| $\bar{\gamma}$ | | *a* | | *b* | | *θ* | | $\overline{\mu}$ | |
| --- | --- | --- | --- | --- | --- | --- | --- | --- | --- |
| True | Mean(MLEs) | True | Mean(MLEs) | True | Mean(MLEs) | True | Mean(MLEs) | True | Mean(MLEs) |
| -1000 | -193.4 | 0.3 | 0.33 | 3333.3 | 586.1 | 2.12 × 10^-4^ | 1.31 × 10^-4^ | 0.1035 | 0.1748 |
| -5000 | -309.2 | 0.3 | 0.35 | 16666.7 | 883.3 | 2.12 × 10^-4^ | 8.86 × 10^-5^ | 0.0639 | 0.1609 |

**Table S4.** Results based on fitting the new models to the INDELs within protein-coding regions from *D. melanogaster*. SNPs from 4-fold sites were used as the neutral reference. But the neutral and the selected regions were assumed to have their separate mutation parameters.

| Model | Parameters for INDELs in the CDS regions | | | | | | | | | ΔAIC |
| --- | --- | --- | --- | --- | --- | --- | --- | --- | --- | --- |
| Continuous | Name | $\theta^{ins}$ | $\alpha^{ins}$ | $b^{ins}$ | $\epsilon^{ins}$ | $\theta^{del}$ | $\alpha^{del}$ | $b^{del}$ | $\epsilon^{del}$ | 0 |
|  | MLE | 8.2 × 10^−5^ | 0.36 | 968.5 | 0.0 | 6.6 × 10^−4^ | 0.57 | 2195.8 | 0.0037 |  |
| Discrete | Name | ${\theta_{1}}^{ins}$ | ${\gamma_{1}}^{ins}$ | ${\epsilon_{1}}^{ins}$ | ${\theta_{1}}^{del}$ | ${\gamma_{1}}^{del}$ | ${\epsilon_{1}}^{del}$ |  |  | 6 |
| *C*=2 | MLE | 1.5 × 10^−5^ | -1.05 | 0.0032 | 3.9 x 10^-5^ | -3.08 | 0.0120 |  |  |  |
|  | Name | ${\theta_{2}}^{ins}$ | ${\gamma_{2}}^{ins}$ | ${\epsilon_{2}}^{ins}$ | ${\theta_{2}}^{del}$ | ${\gamma_{2}}^{del}$ | ${\epsilon_{2}}^{del}$ |  |  |  |
|  | MLE | 6.6 × 10^−5^ | -80.2 | 2.0 x 10^-6^ | 1.8 × 10^−4^ | -56.2 | 2.1 × 10^-6^ |  |  |  |
| Discrete | Name | ${\theta_{1}}^{ins}$ | ${\gamma_{1}}^{ins}$ | ${\epsilon_{1}}^{ins}$ | ${\theta_{1}}^{del}$ | ${\gamma_{1}}^{del}$ | ${\epsilon_{1}}^{del}$ |  |  | 16 |
| *C*=3 | MLE | 1.6 × 10^−5^ | -2.46 | 0.032 | 2.4 × 10^−4^ | -88.4 | 0.0080 |  |  |  |
|  | Name | ${\theta_{2}}^{ins}$ | ${\gamma_{2}}^{ins}$ | ${\epsilon_{2}}^{ins}$ | ${\theta_{2}}^{del}$ | ${\gamma_{2}}^{del}$ | ${\epsilon_{2}}^{del}$ |  |  |  |
|  | MLE | 1.5 × 10^−6^ | 15.8 | 0.0002 | 1.5 × 10^−6^ | 999.9 | 0.4025 |  |  |  |
|  | Name | ${\theta_{3}}^{ins}$ | ${\gamma_{3}}^{ins}$ | ${\epsilon_{3}}^{ins}$ | ${\theta_{3}}^{del}$ | ${\gamma_{3}}^{del}$ | ${\epsilon_{3}}^{del}$ |  |  |  |
|  | MLE | 9.5 × 10^−5^ | -138.0 | 1.4 x 10^-6^ | 4.4 x 10^-5^ | -3.77 | 0.0012 |  |  |  |
| Discrete | Name | ${\theta_{1}}^{ins}$ | ${\gamma_{1}}^{ins}$ | ${\epsilon_{1}}^{ins}$ | ${\theta_{1}}^{del}$ | ${\gamma_{1}}^{del}$ | ${\epsilon_{1}}^{del}$ |  |  | 67 |
| *C*=1 | MLE | 2.6 × 10^−5^ | -2.83 | 0.039 | 9.0 × 10^−5^ | -6.16 | 0.011 |  |  |  |

**Table S5.** Results based on fitting the new models to the INDELs within protein-coding regions from *D. melanogaster*. SNPs from 4-fold sites were used as the neutral reference. The mutation rate ratio between SNPs and INDELs, and that between deletions and insertions, were fixed to 12.2 and 5, respectively (Schrider et al. 2013). Note that only results based on discrete models with *C* = 2 or 3 classes of selected sites are presented. This is because analyses using either a discrete model with *C* = 1 class of sites or a continuous model with *γ* following a reflected gamma distribution failed to converge to biologically meaningful regions of the parameter space. This is probably due to these models are highly unrealistic given the constraints on the mutation rates.

| Model | Parameters for INDELs in the CDS regions | | | | | | | ΔAIC |
| --- | --- | --- | --- | --- | --- | --- | --- | --- |
| Discrete | Name | ${\theta_{1}}^{ins}$ | ${\gamma_{1}}^{ins}$ | ${\epsilon_{1}}^{ins}$ | ${\theta_{1}}^{del}$ | ${\gamma_{1}}^{del}$ | ${\epsilon_{1}}^{del}$ | 0 |
| *C*=2 | MLE | 1.9 × 10^−4^ | -284.1 | 1.2 × 10^−4^ | 0.0010 | -454.8 | 6.2 × 10^−5^ |  |
|  | Name | ${\theta_{2}}^{ins}$ | ${\gamma_{2}}^{ins}$ | ${\epsilon_{2}}^{ins}$ | ${\theta_{2}}^{del}$ | ${\gamma_{2}}^{del}$ | ${\epsilon_{2}}^{del}$ |  |
|  | MLE | 1.6 × 10^−5^ | -1.31 | 0.0092 | 4.9 × 10^−5^ | -3.77 | 0.0082 |  |
| Discrete | Name | ${\theta_{1}}^{ins}$ | ${\gamma_{1}}^{ins}$ | ${\epsilon_{1}}^{ins}$ | ${\theta_{1}}^{del}$ | ${\gamma_{1}}^{del}$ | ${\epsilon_{1}}^{del}$ | 9 |
| *C*=3 | MLE | 2.1 × 10^−5^ | -408.1 | 3.8 × 10^−4^ | 0.0010 | -1009.3 | 0.0195 |  |
|  | Name | ${\theta_{2}}^{ins}$ | ${\gamma_{2}}^{ins}$ | ${\epsilon_{2}}^{ins}$ | ${\theta_{2}}^{del}$ | ${\gamma_{2}}^{del}$ | ${\epsilon_{2}}^{del}$ |  |
|  | MLE | 1.7 × 10^−5^ | -3.25 | 0.0154 | 3.9 × 10^−5^ | -3.03 | 0.0081 |  |
|  | Name | ${\theta_{3}}^{ins}$ | ${\gamma_{3}}^{ins}$ | ${\epsilon_{3}}^{ins}$ | ${\theta_{3}}^{del}$ | ${\gamma_{3}}^{del}$ | ${\epsilon_{3}}^{del}$ |  |
|  | MLE | 2.8 × 10^−6^ | 13.15 | 0.0994 | 8.1 x 10^-5^ | -36.11 | 0.0096 |  |

**Table S6.** Estimates of the mutation rate ratios between SNPs and INDELs and between deletions and insertions.

| Paper | SNP/INDEL | deletions/insertions |
| --- | --- | --- |
| Petrov & Hartl (1998, Mol Biol Evol 15:293-302) | 6.9 | 8.7 |
| Haag-Liautard et al. (2007, Nature 445:82-85) | 4.2 | 3.0 |
| Schrider et al. (2013, Genetics 194:937-953) | 12.2 | 5.0 |

**Table S7.** Results based on fitting the new models to the INDELs within protein-coding regions from *D. melanogaster*. SNPs from 4-fold sites were used as the neutral reference. The mutation rate ratio between SNPs and INDELs, and that between deletions and insertions, were fixed to 6.9 and 8.7, respectively (Petrov & Hartl 1998). Note that only results based on discrete models with *C* = 2 or 3 classes of selected sites are presented. This is because analyses using either a discrete model with *C* = 1 class of sites or a continuous model with *γ* following a reflected gamma distribution failed to converge to biologically meaningful regions of the parameter space. This is probably due to these models are highly unrealistic given the constraints on the mutation rates. The likelihood surface for the *C* = 3 model also appears to be somewhat flat – a parameter combination without large *γ* values only has a slightly lower log likelihood (516951.999 versus 516951.842).

| Model | Parameters for INDELs in the CDS regions | | | | | | | ΔAIC |
| --- | --- | --- | --- | --- | --- | --- | --- | --- |
| Discrete | Name | ${\theta_{1}}^{ins}$ | ${\gamma_{1}}^{ins}$ | ${\epsilon_{1}}^{ins}$ | ${\theta_{1}}^{del}$ | ${\gamma_{1}}^{del}$ | ${\epsilon_{1}}^{del}$ | 0 |
| *C*=2 | MLE | 2.1 × 10^−4^ | -310.0 | 7.3 × 10^−5^ | 0.0019 | -909.7 | 1.0 × 10^−4^ |  |
|  | Name | ${\theta_{2}}^{ins}$ | ${\gamma_{2}}^{ins}$ | ${\epsilon_{2}}^{ins}$ | ${\theta_{2}}^{del}$ | ${\gamma_{2}}^{del}$ | ${\epsilon_{2}}^{del}$ |  |
|  | MLE | 1.6 × 10^−5^ | -1.29 | 0.0094 | 4.9 × 10^−5^ | -3.81 | 0.0083 |  |
| Discrete | Name | ${\theta_{1}}^{ins}$ | ${\gamma_{1}}^{ins}$ | ${\epsilon_{1}}^{ins}$ | ${\theta_{1}}^{del}$ | ${\gamma_{1}}^{del}$ | ${\epsilon_{1}}^{del}$ | 8 |
| *C*=3 | MLE | 1.6 × 10^−5^ | -2.93 | 5.4 × 10^−4^ | 1.3 × 10^−6^ | 1000 | 0.0012 |  |
|  | Name | ${\theta_{2}}^{ins}$ | ${\gamma_{2}}^{ins}$ | ${\epsilon_{2}}^{ins}$ | ${\theta_{2}}^{del}$ | ${\gamma_{2}}^{del}$ | ${\epsilon_{2}}^{del}$ |  |
|  | MLE | 2.3 × 10^−4^ | -394.7 | 3.5 × 10^−4^ | 5.2 × 10^−5^ | -4.61 | 1.3 × 10^−4^ |  |
|  | Name | ${\theta_{3}}^{ins}$ | ${\gamma_{3}}^{ins}$ | ${\epsilon_{3}}^{ins}$ | ${\theta_{3}}^{del}$ | ${\gamma_{3}}^{del}$ | ${\epsilon_{3}}^{del}$ |  |
|  | MLE | 2.9 × 10^−6^ | 1000 | 0.1473 | 0.0021 | -1046.9 | 0.0292 |  |

**Table S8.** Results based on fitting the new models to the INDELs within protein-coding regions from *D. melanogaster*. SNPs from 4-fold sites were used as the neutral reference. The mutation rate ratio between SNPs and INDELs, and that between deletions and insertions, were fixed to 4.2 and 3, respectively (Haag-Liautard et al. 2007). Note that only results based on discrete models with *C* = 2 or 3 classes of selected sites are presented. This is because analyses using either a discrete model with *C* = 1 class of sites or a continuous model with *γ* following a reflected gamma distribution failed to converge to biologically meaningful regions of the parameter space. This is probably due to these models are highly unrealistic given the constraints on the mutation rates.

| Model | Parameters for INDELs in the CDS regions | | | | | | | ΔAIC |
| --- | --- | --- | --- | --- | --- | --- | --- | --- |
| Discrete | Name | ${\theta_{1}}^{ins}$ | ${\gamma_{1}}^{ins}$ | ${\epsilon_{1}}^{ins}$ | ${\theta_{1}}^{del}$ | ${\gamma_{1}}^{del}$ | ${\epsilon_{1}}^{del}$ | 0 |
| *C*=2 | MLE | 8.9 × 10^−4^ | -1451.6 | 4.1 × 10^−5^ | 0.0027 | -1292.7 | 3.0 × 10^−5^ |  |
|  | Name | ${\theta_{2}}^{ins}$ | ${\gamma_{2}}^{ins}$ | ${\epsilon_{2}}^{ins}$ | ${\theta_{2}}^{del}$ | ${\gamma_{2}}^{del}$ | ${\epsilon_{2}}^{del}$ |  |
|  | MLE | 1.7 × 10^−5^ | -1.45 | 0.0104 | 5.0 × 10^−5^ | -3.89 | 0.0083 |  |
| Discrete | Name | ${\theta_{1}}^{ins}$ | ${\gamma_{1}}^{ins}$ | ${\epsilon_{1}}^{ins}$ | ${\theta_{1}}^{del}$ | ${\gamma_{1}}^{del}$ | ${\epsilon_{1}}^{del}$ | 8 |
| *C*=3 | MLE | 1.6 × 10^−5^ | -2.72 | 0.0046 | 3.8 × 10^−5^ | -2.98 | 0.0065 |  |
|  | Name | ${\theta_{2}}^{ins}$ | ${\gamma_{2}}^{ins}$ | ${\epsilon_{2}}^{ins}$ | ${\theta_{2}}^{del}$ | ${\gamma_{2}}^{del}$ | ${\epsilon_{2}}^{del}$ |  |
|  | MLE | 2.4 × 10^−6^ | 13.69 | 0.14 | 0.0028 | -5548.2 | 0.0288 |  |
|  | Name | ${\theta_{3}}^{ins}$ | ${\gamma_{3}}^{ins}$ | ${\epsilon_{3}}^{ins}$ | ${\theta_{3}}^{del}$ | ${\gamma_{3}}^{del}$ | ${\epsilon_{3}}^{del}$ |  |
|  | MLE | 9.5 × 10^−4^ | -1763.1 | 3.4 × 10^−5^ | 1.3 × 10^4^ | -43.92 | 0.0045 |  |

**Table S9.** A comparison of parameter estimates obtained from analyses based on different mutation rate ratio estimates.

| Source of mutation rate ratios | Weakly selected sites | | *α* (%) | | |
| --- | --- | --- | --- | --- | --- |
|  | *γ*­*^ins^* | *γ*­*^del^* | INDELs | insertions | deletions |
| Petrov & Hartl (1998) | -1.29 | -3.81 | 71.6 | 59.5 | 81.6 |
| Haag-Liautard et al. (2007) | -1.45 | -3.89 | 72.9 | 61.5 | 82.3 |
| Schrider et al. (2013) | -1.31 | -3.77 | 71.5 | 59.7 | 81.3 |
