# Supplementary figures and images for: New Methods for Inferring the Distribution of Fitness Effects for INDELs and SNPs
Source: Mol Biol Evol. 2018 Apr 4;35(6):1536–46. doi: 10.1093/molbev/msy054 (PMC5967470; doi:10.1093/molbev/msy054)

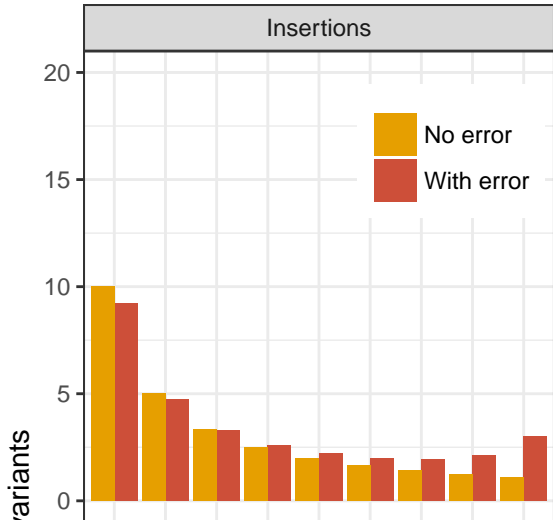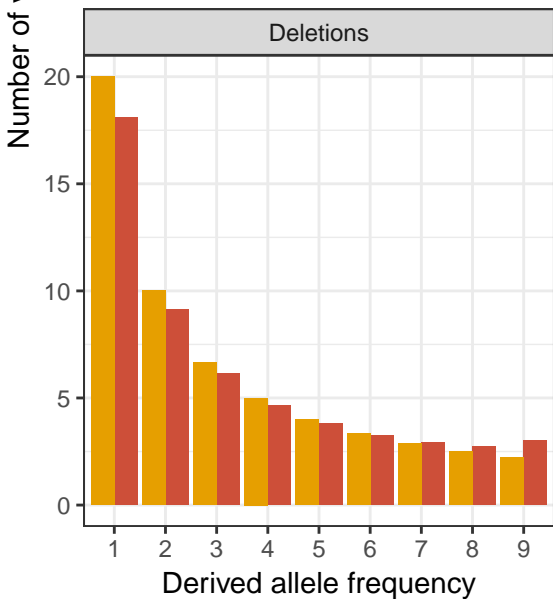

Supplement: Supplementary Data [file msy054_supp.zip › fig1.pdf]
